# The impact of general pedagogical knowledge on student engagement as mediated by instructional communication

**DOI:** 10.1038/s41598-026-60556-0

**Published:** 2026-07-07

**Authors:** Workineh Birhanu Aynalem, Tiruwork Tamiru Tolla, Amare Sahile Abebe

**Affiliations:** Department of Psychology, School of Educational Sciences, College of Education, Bahir Dar University, Bahir Dar, Ethiopia

**Keywords:** Ethiopia, Instructional communication, Mediation analysis, Pedagogical knowledge, Student engagement, Teacher education, Ecology, Psychology

## Abstract

Teachers’ ability to apply pedagogical knowledge through effective classroom communication is an important factor in shaping student engagement, yet this pathway remains underexplored in Ethiopian higher education. This study examined how general pedagogical knowledge (GPK) relates to student engagement (SE) through the process role of instructional communication (IC). Using a quantitative explanatory design, data were collected from 278 teachers across ten Ethiopian colleges and analyzed with structural equation modeling (SEM). Three validated instruments measured GPK, IC, and SE based on teachers’ perceptions. Results showed that GPK had a significant effect on IC (β = 0.26, *p* = .013), and IC strongly predicted SE (β = 0.82, *p* < .001). The direct effect of GPK on SE was not significant, indicating that IC functioned as the process variable fully linking GPK to SE. These findings suggest that teachers’ pedagogical knowledge supports engagement primarily through communicative clarity and responsiveness. However, because engagement was assessed only through teacher self-reports, the results should be interpreted as perceptions rather than direct measures of student behavior. The study contributes to understanding the GPK–IC–SE pathway and highlights the need for teacher education programs to strengthen communicative competence alongside pedagogical expertise.

## Introduction

Teaching quality depends on both subject specific knowledge (pedagogical content knowledge, PCK) and general pedagogical knowledge (GPK). While PCK links subject matter to teaching methods, GPK refers to general teaching skills such as managing classrooms, motivating students, assessing learning, and structuring lessons^1,2^. Building on Shulman’s framework, Mishra and Koehler’s TPACK model highlights the importance of communication as the bridge between pedagogical knowledge and student engagement^3^.

Instructional communication (IC) refers to how teachers convey content, manage interactions, and shape classroom climate. Effective IC-characterized by clarity, immediacy, and responsiveness—strengthens instructional quality and student engagement (SE)^4,5^. Student engagement (SE), comprising behavioral, emotional, and cognitive dimensions, is a key outcome of effective teaching and communication, fostering persistence and deeper learning^6,7^.

Although GPK is widely recognized as essential, its pathway to SE through IC has received limited empirical attention, particularly in Ethiopian higher education. Existing studies often examine GPK, IC, or SE separately. Few yet tested their interrelationships only without interaction^8,9^. This gap limits both theoretical understanding of the field and practical guidance for teacher education programs.

This study addresses this gap by testing whether IC functions as the process variable through which teachers’ GPK influences SE. Using structural equation modeling (SEM) with data from 278 instructors, we estimate the GPK → IC, IC → SE, and GPK → SE paths to determine whether mediation is full or partial. The study contributes theoretically by clarifying how pedagogical knowledge is enacted through communicative processes, and practically by informing teacher education programs seeking to strengthen communicative competence alongside pedagogical expertise.

## Literature Review

Pedagogical knowledge remains central to teaching quality, but contemporary research distinguishes between subject specific pedagogical content knowledge (PCK) and general pedagogical knowledge (GPK). GPK encompasses cross disciplinary competencies such as classroom management, lesson planning, assessment, and student motivation, forming the foundation for effective instruction across contexts^1,2^. Building on this, Mishra and Koehler’s^3^ Technological Pedagogical Content Knowledge (TPACK) framework—and its recent extensions to contextual knowledge^10,11^—emphasize that effective teaching requires integration of pedagogy, content, and technology, with communication serving as the bridge between knowledge and practice.

Instructional communication (IC) refers to teachers’ verbal and non-verbal behaviors that shape classroom climate and interaction. Recent studies confirm clarity, immediacy, responsiveness, and feedback as predictors of student engagement (SE)^7,12^. Engagement itself is multidimensional—behavioral, emotional, cognitive, and agentic—and consistently linked to persistence and deeper learning outcomes^6,13^. Meta analyses further demonstrate that IC often mediates the relationship between teaching quality and student outcomes, with socio emotional competence emerging as a critical factor^14,15^. Importantly, IC should be understood not only as a mediator but as a process variable through which pedagogical knowledge is enacted and translated into engagement.

In Ethiopia, emerging studies reveal persistent challenges in fostering engagement, including reliance on lecture-based delivery and limited modeling of communicative strategies. Surveys and multilevel analyses show that teacher support, self-efficacy, and classroom level practices explain significant variance in perceived engagement, yet mediation models remain scarce^16,17^. This gap underscores the need to examine how GPK influences SE through IC in Ethiopian higher education, situating local findings within the broader global discourse on teaching quality and engagement.

### Link to conceptual framework

**Fig. 1:**
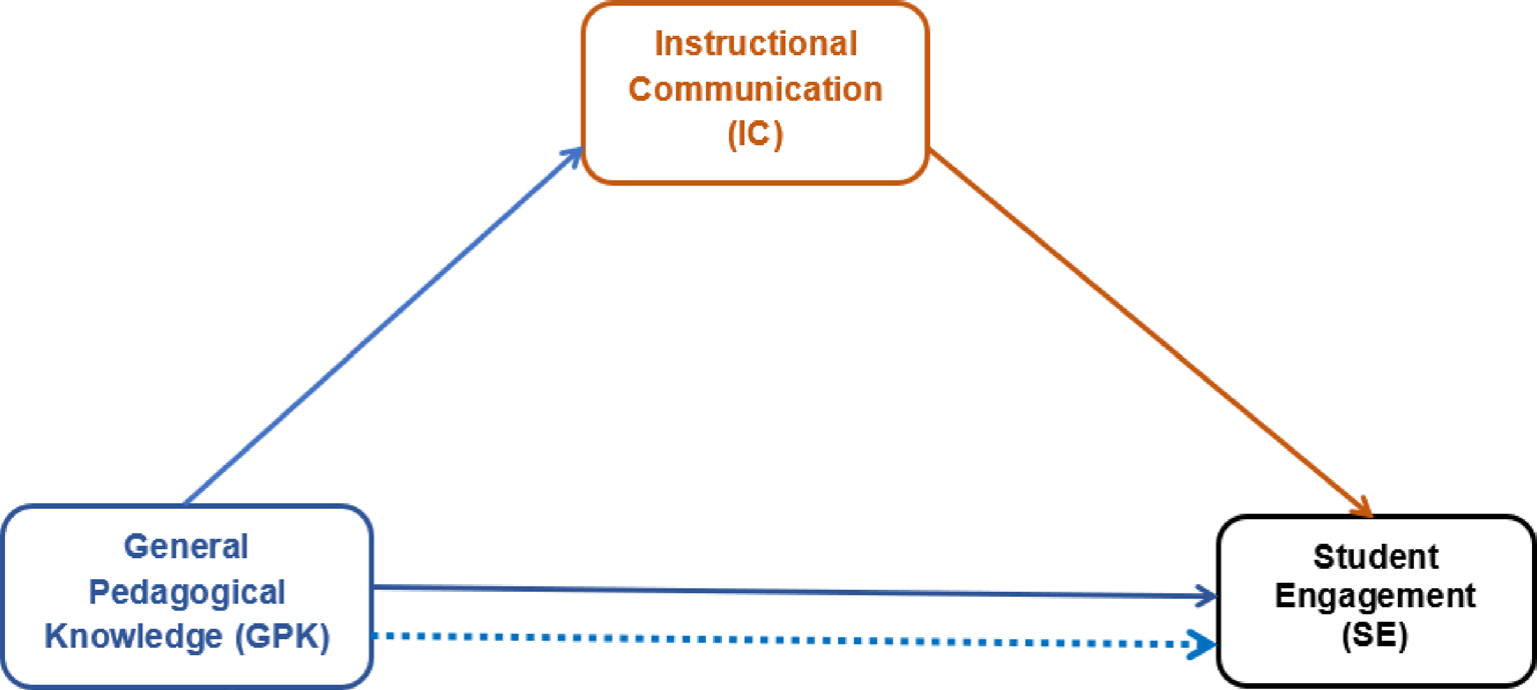
Conceptual framework, illustrating IC as the process variable linking GPK to SE.

These insights underpin the present study’s conceptual framework, in which Instructional Communication is hypothesized to mediate the relationship between General Pedagogical Knowledge and Student Engagement. The next section sets out this framework, elaborating the proposed pathways and associated hypotheses as depicted in Fig. 1.

The study proposes a conceptual model in which General Pedagogical Knowledge (GPK) shapes Student Engagement (SE) through Instructional Communication (IC), positioned as the process variable that enacts pedagogical expertise.

## Methods

### Research design

This study employed a quantitative explanatory design using structural equation modeling (SEM) to examine the hypothesized relationships among general pedagogical knowledge (GPK), instructional communication (IC), and student engagement (SE).

### Participants and Sampling

The population consisted of 902 teachers from ten Ethiopian colleges. A stratified random sample of 278 teachers was selected to ensure representation across sex, years of service, qualifications, and teaching experience.

### Measures

Three validated instruments were adapted for local relevance:

- ***General Pedagogical Knowledge (GPK)***: 28 items across six dimensions, adapted from the Teacher Knowledge Survey. Reliability ranged from α = 0.836–0.930.
- ***Instructional Communication (IC)***: 16 items across three dimensions, adapted from the Teacher–Student Instructional Communication Scale. Reliability ranged from α = 0.718–0.844.
- ***Student Engagement (SE)***: 21 items across four dimensions, adapted from the Student Engagement Instrument–College. Reliability ranged from α = 0.725–0.919.

All instruments used five-point Likert scales. Teachers completed the questionnaires in English, the medium of instruction in Ethiopian colleges.

### Data Collection and Preparation

Data were screened for missing values, outliers, and normality. Items with low factor loadings (< 0.40) were removed. Confirmatory factor analyses (CFA) confirmed construct validity, with acceptable fit indices across all three measures.

### Ethical Considerations

Ethical approval was obtained from Bahir Dar University Institutional Research Review Committee (BDU-IRRC). Participation was voluntary, with informed consent secured. Confidentiality and anonymity were maintained in line with APA ethical standards and the Declaration of Helsinki.

### Limitations of Measurement

It is important to note that student engagement was measured exclusively through teachers’ perceptions. While this approach provides valuable insights into teachers’ views, it does not capture students’ actual engagement behaviors. This limitation should be considered when interpreting the findings, and future research should incorporate student self-reports, classroom observations, or objective indicators to strengthen validity.

## Results

Confirmatory factor analyses supported the construct validity of GPK, IC, and SE. All retained items loaded above 0.50, and fit indices (CFI > 0.90, RMSEA < 0.06) indicated acceptable validity.

Structural equation modeling showed:

- GPK → IC: modest positive effect (β = 0.26, *p* =.013).
- IC → SE: strong positive effect (β = 0.82, *p* <.001).
- GPK → SE (direct): small, not significant (β = 0.10, *p* =.254).
- Indirect effect (GPK → IC → SE): significant mediation (β = 0.22, *p* <.001).

**Fig. 2:**
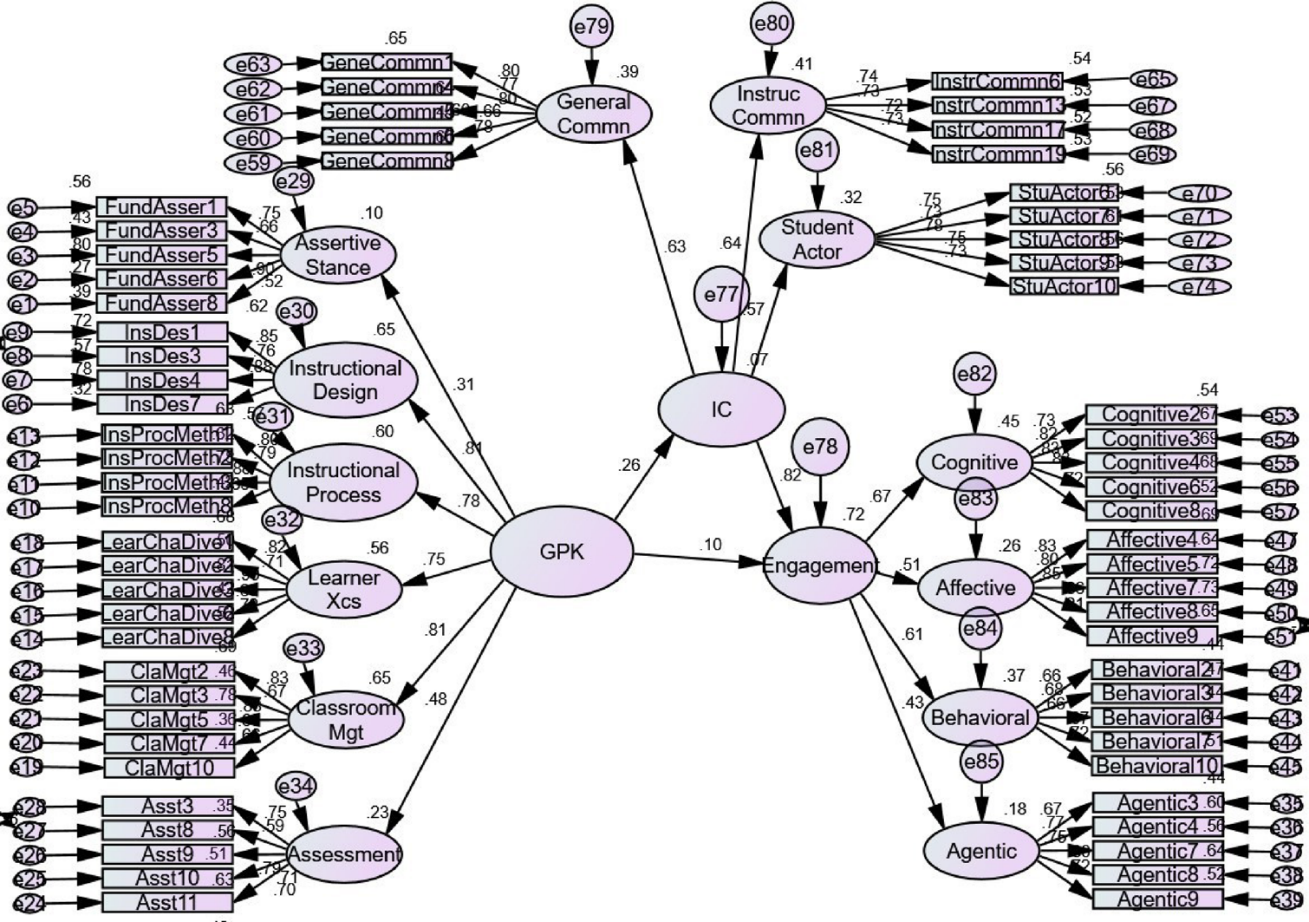
Research model of the study.

The final structural model is presented see Fig. 2.

The model showed that GPK explained 6.7% of the variance in IC, while IC explained 71.7% of the variance in SE. Thus, IC served as the central process variable linking GPK to SE. However, the modest variance explained in IC indicates that other factors could have also shaped teachers’ communicative practices. Summary of the path coefficients is also showed in Table 1.

**Table 1:** Structural path estimates of mediation.

Hypothesized Relationship	Path	Std. Estimate	95% CI	*p*-value	*R*²
GPK → IC	a	0.26	[0.16, 0.36]	0.013	0.067
IC → SE	b	0.82	[0.72, 0.92]	< 0.001	0.717
GPK → SE (Direct)	c′	0.10	[0.00, 0.20]	0.254	–
Indirect (GPK → IC → SE)	a*b	0.22	[0.12, 0.32]	< 0.001	–
Total Effect	c	0.32	[0.22, 0.42]	0.015	–

Note: 95% CI based on 5,000 bootstrap samples.

## Discussion

This study examined how GPK relates to SE through the process role of IC. Findings suggest that while GPK contributes to teachers’ communicative practices, IC is the process variable most strongly associated with perceived engagement. Although mediation was statistically significant, the modest variance explained in IC means the strength of this pathway should be interpreted cautiously.

Pedagogical knowledge may support engagement primarily by enabling teachers to communicate more clearly and responsively. Yet knowledge alone is insufficient; IC is the process through which expertise is enacted. This aligns with meta analyses showing teacher clarity and immediacy predict engagement, and with studies highlighting socio emotional competence as a mediating factor. This aligns with recent meta analyses showing that teacher clarity, immediacy, and responsiveness are consistent predictors of engagement across higher education contexts^7,12^. Similarly, Keery and Gemeda^14^ found that socio emotional competence mediates the relationship between teaching quality and student outcomes.

**Fig. 3:**
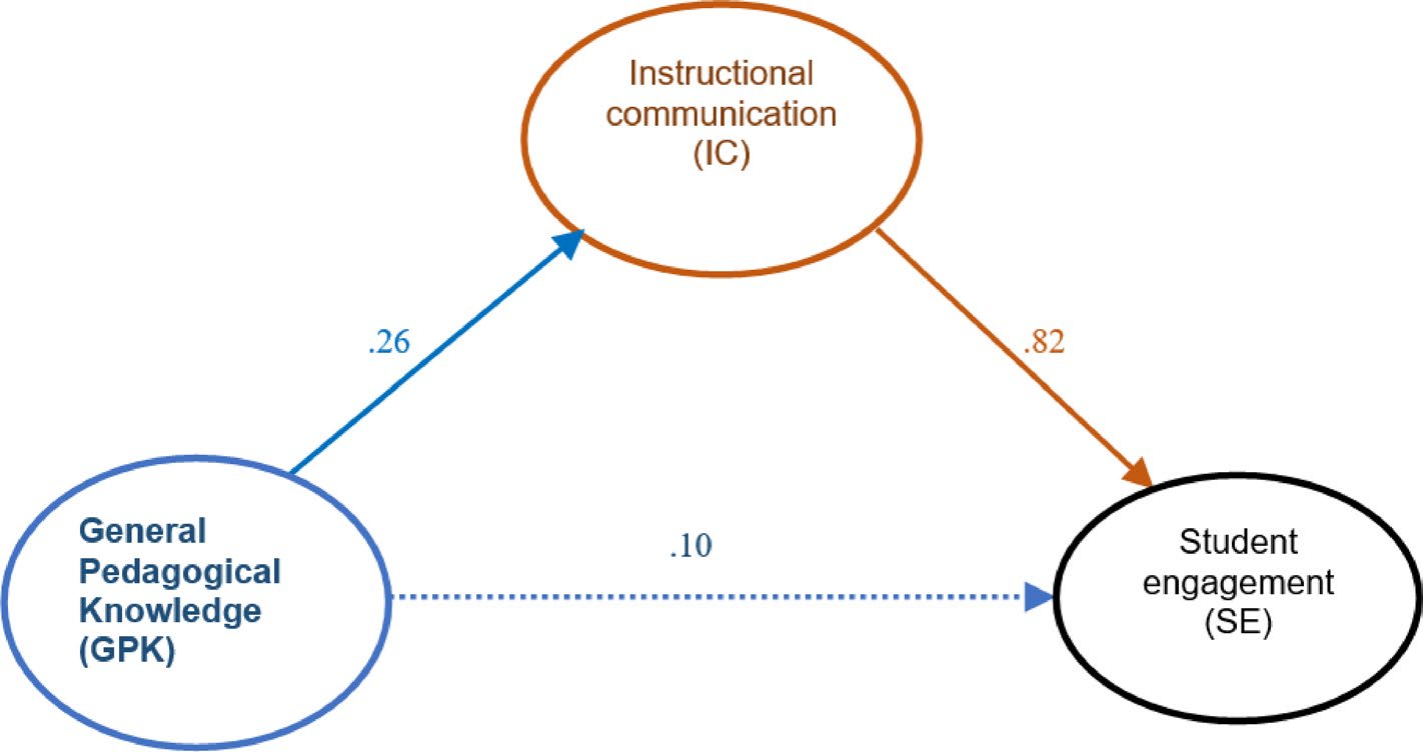
Theoretical model of the study.

The findings also resonate with theoretical perspectives such as the TPACK framework^3,11^, though not focused in the study by far. In this sense, IC serves as the bridge between knowledge and practice. This in turn supports the notion that socio constructivist views of teaching as dialogic interaction^9^. Hence, the user‑defined theoretical model, illustrates the relationships among the three major factors (see Fig. 3).

The study advances a conceptual framework where General Pedagogical Knowledge (GPK) influences Student Engagement (SE) by means of Instructional Communication (IC). In this model, IC is explicitly framed as the process variable through which pedagogical knowledge is enacted and translated into engagement.

Educationally, the results suggest that teacher education programs should integrate explicit training in instructional communication alongside pedagogical knowledge. However, the causal impact should be interpreted carefully, given the modest variance explained and reliance on perceptual data^18,19^. Teacher education programs may therefore benefit from integrating explicit training in instructional communication alongside pedagogical knowledge.

### The context

In Ethiopia, recent studies highlight persistent challenges in fostering engagement, including reliance on lecture-based delivery and limited modeling of communicative strategies^17^. Surveys at Mattu University show that self-efficacy, teacher support, and technology support significantly predict engagement (Faro et al., 2025), while multilevel analyses confirm that classroom level practices explain substantial variance in student outcomes^20^. The present findings extend this literature by demonstrating that GPK’s influence on engagement is indirect, operating through IC. This condition emphasizes the need for Ethiopian teacher education programs to emphasize communicative competence as a core dimension of pedagogical training.

### Limitations and future research

A key limitation is that SE was measured only through teachers’ perceptions. These perceptions provide valuable insights but may not fully reflect students’ actual engagement behaviors. Without triangulation using student self-reports, classroom observations, or objective indicators, the findings should be interpreted as perceptions rather than direct measures. This limitation also shapes the implications: future research should employ mixed method or student-centered measures to extend the present model and provide a more comprehensive understanding of engagement. Testing mediation models across diverse contexts would further clarify how IC functions as a process variable.

## Conclusion

This study provides new evidence on how GPK influences SE through IC in Ethiopian higher education. While GPK contributed modestly to teachers’ communicative practices, IC emerged as the central process variable linking pedagogical expertise to perceived engagement. These findings highlight the importance of communication as the bridge between knowledge and practice, but they should be interpreted with caution given the modest variance explained and reliance on teacher self-reports.

The study advances theoretical understanding of the GPK–IC–SE pathway and underscores the need for teacher education programs to integrate communicative competence alongside pedagogical expertise. Practically, strengthening teachers’ clarity, responsiveness, and immediacy may enhance engagement, though these implications remain tentative until validated with broader measures. In sum, pedagogical knowledge alone is insufficient; it is through communication that knowledge becomes engagement.

## Data Availability

No datasets were generated or analysed during the current study.
